# Flesh ID: Nanopore Sequencing Combined with Offline BLAST Search for the Identification of Meat Source

**DOI:** 10.3390/foods9101392

**Published:** 2020-10-01

**Authors:** Jonas Kissenkötter, Susanne Böhlken-Fascher, Ahmed Abd El Wahed

**Affiliations:** 1 Department of Animal Science, Division of Microbiology and Animal Hygiene, Georg-August-University of Goettingen, D-37077, Goettingen, Germany; susanne.boehlken-fascher@agr.uni-goettingen.de (S.B.-F.); ahmed.abd_el_wahed@uni-leipzig.de (A.A.E.W.); 2 Institute of Animal Hygiene and Veterinary Public Health, University of Leipzig, D-04103, Leipzig, Germany

**Keywords:** meat adulteration, rapid sequencing protocol, nanopore sequencing, point-of-need

## Abstract

Detection of animal species in meat product is crucial to prevent adulterated and unnecessary contamination during processing, in addition to avoid allergy and religious consequences. Gold standard is the real-time PCR assays, which has a limited target capability. In this study, we have established a rapid sequencing protocol to identify animal species within hours. Sequencing was achieved by nanopore sequencing and data analysis *via* offline BLAST search. The whole procedure was conducted in a mobile suitcase lab. As per national and international regulations, the developed assay detected adulteration of pork meat with 0.1% of horse, chicken, turkey, cattle, sheep, duck, rabbit, goat, and donkey. The developed test could be used on-site as a rapid and mobile detection system to determine contamination of meat products.

## 1. Introduction

Un- or incorrectly declared species in food can lead to considerable health risks or attack religious taboos [1]. In addition to the prohibition of some animal species from consumption in some religions, certain animal species pose a high health risk for consumers [2]. Adulteration of meat products with exotic meat increases the risk of introducing emerging infectious diseases. On top of that, many incidents of mixing expensive type of meat with poor one were reported [3]. In the horse meat scandal in 2013, the food processing industry processed horse meat and offered it incorrectly declared to customers for sale [4]. The fundamental problem remains that the inspection of meat that is supplied or processed is posing increasing challenges to the food industry and to official food control [5]. So far, the meat origin can only be clarified by very specific analytical methods such as immunological assays or DNA-based amplification technologies [6,7]. The mass spectrometry has emerged as a rapid and accurate method for the identification of meat source [8,9]. The gold-standard in the authentication process of meat and meat products is the real-time PCR [10]. Recently, many isothermal amplification assays were established [11,12]. However, these molecular tests are limited to few targets and are unable to identify species outside the narrow target range. As a consequence, delay in diagnosis, false negative, and increase costs may occur. Metagenomic sequencing is a promising solution to overcome these limitations [13]. A number of assays relying on high throughput second generation or Next Generation Sequencing (NGS) technologies were developed to detect and genetically characterize animal species [14,15]. However, challenges remain with dependence on PCR based amplification, cumbersome end-point result analysis, logistic demand, cost, applicability in field site, and restriction to laboratory settings. Fourth generation sequencing such as nanopore technology confers a promising alternative to offer feasible, field deployable, and rapid sequencing option with a real-time data acquisition [16,17]. The technology was applied for identification of fish species, but the bioinformatics remained a great obstacle [18]. Therefore, we are aiming to evaluate the performance of this metagenomic sequencing based on nanopore technology in detecting animal species in meat, as well as developing user friendly offline-BLAST search for data analysis.

## 2. Materials and Methods

### 2.1. Meat Samples

**Figure 1:**
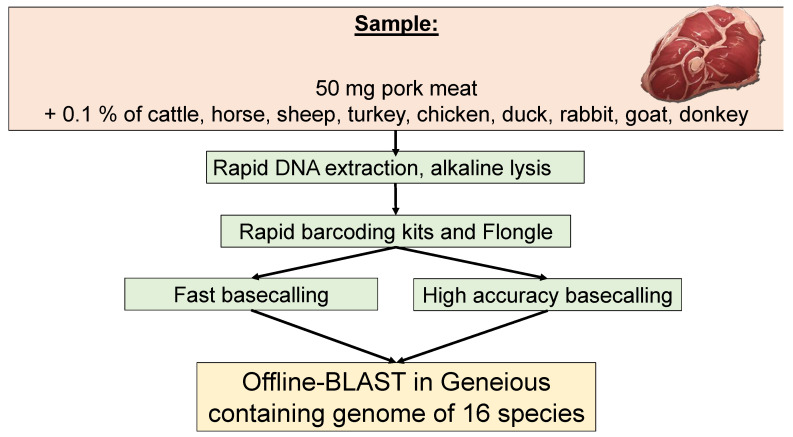
Flesh ID experiment workflow.

Vacuum-packed meat of pig, cattle, sheep, horse, chicken, turkey, duck and rabbit was purchased from a local supermarket. The standard genomic DNA of goat and donkey were provided by Eurofins GeneScan Technologies GmbH (Freiburg, Germany). According to the German Federal Office of Consumer Protection and Food Safety, 0.1% considered as the lower limit of detection of meat contamination (German Food and Feed Code §64 (LFGB)) [12], therefore, using a sensitive balance, 50 mg of pork meat was spiked with 0.5 mg of cattle, sheep, horse, chicken, turkey, duck, and rabbit meat. Since, meat source from goat and donkey were not available, genomic DNA of both species equivalence to 10^3^ molecules were added to the mix. Total nucleic acid from the meat mixture was extracted by using an alkaline lysis protocol adjusted from Girish et al. [19]. Briefly, a total of 200 µL lysis buffer (200 mM NaOH) was applied to the meat sample and incubated for one hour at room temperature; thereafter, a neutralization step was conducted by adding 400 µL of Tris-HCL (0.04 M pH 7.5) to the meat sample lysis buffer mix. The amount of DNA was measured by using the Qubit 2.0 Fluorometer (Invitrogen, Carlsbad, CA, USA). The sample was adjusted to a DNA amount of 200 ng/3.75 µL for sequencing. One sample containing only pig genomic material was used as a background control. Figure 1 is a schematic presentation of the study procedure. 

### 2.2. Sequencing Library Preparation 

For library preparation, the rapid sequencing kit (SQK-RAD004) and Flongle from Oxford Nanopore technologies (Cambridge, UK) were used. A total of 200 ng DNA of the meat mixture was incubated with rapid adapters at 30 °C for one minute. During the incubation, the DNA was fragmented with the Transposon and the sequencing adaptors and barcodes were attached. To avoid unnecessary Transposon’s activities and the production of very short DNA fragment, the mixture was incubated at 80 °C for one minute. The sequencing buffer and loading beads were prepared as instructed by the manufacturer. It is important to mention that the loading style of the mix to the Flongle must be conducted by attaching the filter tips of 200 µL automatic pipette to the sample port, then rotating the volume adjustment knob in clockwise manner. Pushing the fluid using the plunger can destroy the nanopore membrane [20]. 

### 2.3. Sequencing

Sequencing was conducted on the MinION device including both Flongle adaptor and cell. Data acquisition and basecalling were carried out in real-time by the MinKNOW software. The equipment and software were purchased or downloaded from Oxford Nanopore technologies (Cambridge, UK). Sequencing was performed for up to 48 h using −180 Voltage.

### 2.4. Data Analysis 

For data analysis, all generated data files in Fastq-format were transferred to the Software Geneious 10.2.3. Here, the sequences of all available chromosomes of Horse, Chicken, Turkey, Cattle, Sheep, Duck, Rabbit and Goat were downloaded from the NCBI database (Table 1). For Donkey, only shotgun reference was available (GCA_001305755.1) and for the Pig, sequence with accession number: NC_010443.5 was used. The accuracy of the selected database for the offline BLAST-search was tested by using reference sequences of six additional animal species (dog, NC_002008.4; impala, NC_020675; lion, CM_018460.1; bison, NC_12346; camel, NC_009849.1; Japanese quail, NC_003408.1). The speed of species identification was measured by analyzing sequence data generated after 0.5, 1, 3, and 9 h of the sequence run. 

## 3. Results

### 3.1. Data Acquisition

**Figure 2:**
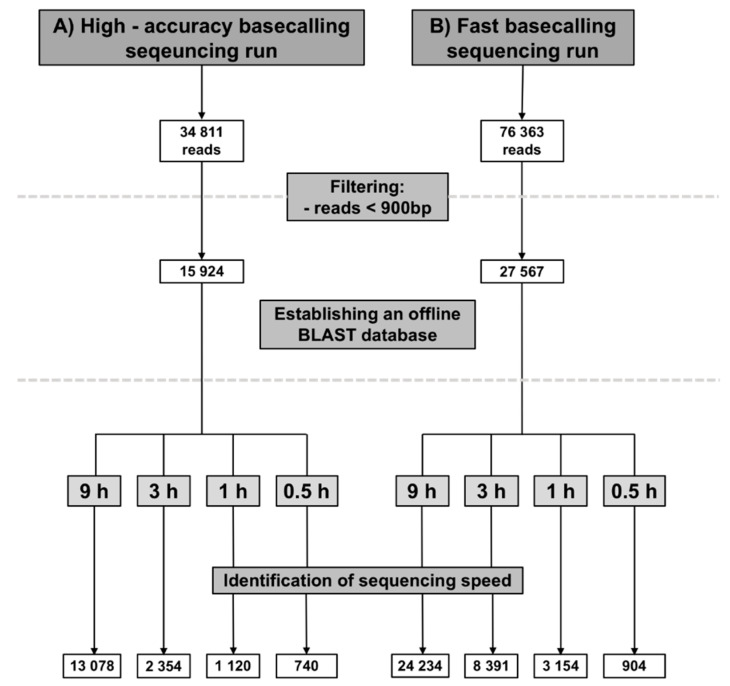
Number of reads of the high-accuracy (**A**) and fast (**B**) basecalling runs in total, after filtering and at various time points.

The MinKnow software saved Fastq sequence files directly on the computer hard desk. All raw sequence data files are freely available for public on https://doi.org/10.5281/zenodo.4034907. In total, 34,811 reads were collected after 48 h of the high-accuracy sequencing run. All reads with a length lower than 900 bases were deleted to avoid the inclusion of short inaccurate sequences (Figure 2). One very important issue, the "What’s in my pot" of the Epi2Me software (Oxford Nanopore technologies, Cambridge, UK) did not identify any of the meat species. Therefore, the establishment of an offline-BLAST search using Geneious software was necessary.

### 3.2. Offline BLAST-Search

**Figure 3:**
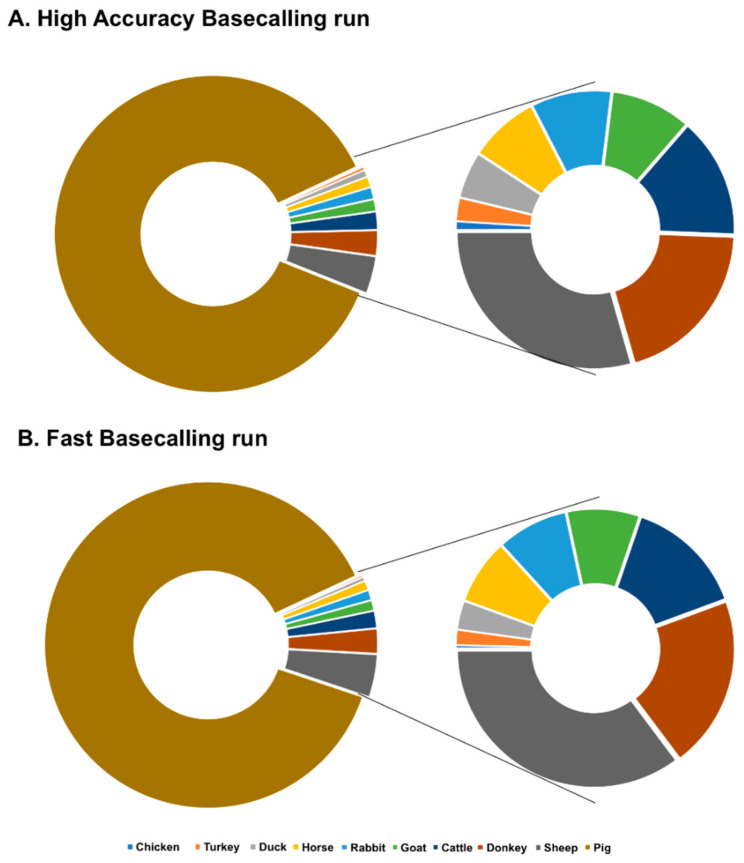
Results of the offline BLAST-search by applying the Flesh ID database to the sequencing data of the high-accuracy (**A**) and fast (**B**) basecalling run. In both sequence runs, all 10 animal species could be identified using the Flesh ID database.

The filtered reads of the high-accuracy run were blasted against all selected sequences (Table 1). For the BLAST-search, the fast and high similarity Megablast program were chosen, with only a Query-centric alignment and a maximum of one hit per read. The e-value was set to 1 × 10^−100^. All expected animal species could be identified (Figure 3; Table 1). As anticipated, the highest number of hits were assigned to the pig reference sequence, while the poultry species produced lower number of hits than the mammal species. No possible explanation was found for the lower hit numbers by Chicken. 

### 3.3. Identification of Sequencing Speed

For identification of the sequencing run duration, the reads produced within the first 30 min, one, three, and nine hours of the high-accuracy basecalling were analyzed. Surprisingly, the reads produced in the first hour against chromosome one of pig, sheep, goat, horse, and duck, and chromosome 2 of chicken and cattle, chromosome 3 of turkey, and chromosome 7 of rabbit were sufficient to identify all ten animal species in the meat mixture. The pairwise identity between the hits and the corresponding reference sequences ranged from 80.33 to 85.96% (Table 2).

To validate the results, the sequence run was repeated using the fast basecalling model. In total 76,363 reads were obtained. All species was identified within one hour of sequencing except chicken was detected first after 9 h (Table 2).

### 3.4. Database Specificity

To assess the accuracy of the database to correctly identify the possible meat adulteration, the whole reference sequence of the mitochondrial genome of five unrelated animal species (dog, impala, camel, bison, and the Japanese quail) and one shotgun sequence of the lion genome were chosen as a negative database. No hits were assigned to these sequences by performing an offline BLAST-search with this database on the sequence reads of the high-accuracy and fast basecalling runs. This indicates high specificity of the offline BLAST-search. The sequence reads (total: 31,344) of sample containing only background pig genomic materials have assigned only to the reference sequence of *Sus scrofa* and did not assorted to the other animal species, which again indicate the accuracy of the offline-BLAST search. 

## 4. Discussion

For the identification of animal species in meat products, nanopore sequencing was combined with an offline BLAST-search. The DNA was extracted in one hour using alkaline lysis buffer. Library preparation was conducted in 10 min and the sequencing run in up to 48 h. The offline BLAST-search in Geneious was achieved in less than 20 min. 

Oxford nanopore developed two basecalling options, the high-accuracy (Flip-flop basecalling) and the fast model. While the high-accuracy basecalling produces a higher raw read quality with a basecalling speed of 4.4 k bases/s, the fast model has a speed of 36 k bases/s, which results in a lower raw read accuracy [21]. In our experiment, the double number of the reads was collected by the fast basecalling, but both methods produced similar sequence accuracy (Figure 3; Table 2). Nevertheless, the data of both basecalling method lead to the identification of all animal species in the meat mixture. The only difference was the speed as all species were identified after one hour in the high-accuracy sequencing run, while nine hours was needed for the fast basecalling (Table 2) as all genome from all animals except chicken behaved the same. We did not find possible reason of the lower performance of chicken genome in nanopore sequencing, which remain a big question mark. 

Oxford nanopore offers a range of online data analyzing tools. Sequencing data can be uploaded to the cloud-based *Epi2Me* platform for real-time analysis workflows [22]. Unfortunately, only virus, bacteria, fungi, and archaea sequences can be recognized by *Epi2Me*. Therefore, for the identification of animal species in meat mixtures, the offline BLAST-search Flesh ID database was developed and data analysis was achieved in Geneious software. In our database, reference sequences of various chromosomes of the animal species were included (Table 1). In other studies, specific genes were selected for the identification process. Most commonly mitochondrial genes like the COI [23], the cytb [24], the 16S [25] and the 12S gene [26]. The authors performed an amplification step using PCR before sequencing, which resulting in a more complex and time-consuming library preparation. Using nanopore sequencing combined with offline-BLAST search, whole genome sequencing is possible as no amplification step is needed during library preparation. Another advantage is the use of portable sequencing device, the MinION, which can easily be implemented at point of need [27]. The data analysis and species identification can be performed by an online BLAST tool against all possible odds, which, off course, will increase the chances of detecting exotic species. Nevertheless, a powerful internet connection and hardware are required, moreover, the run time will exceed many hours in case of analyzing thousands of nanopore long sequencing reads. On other hand, the offline BLAST search limited to the reference sequences of animal species included in the local database, but can be extended according to the end-user needs. 

More than three quarters of the sequence reads belongs to the host genes and the rest is the microbiome. In our study, the huge amount of host sequence is an advantage since the aim to identify the animal species. Nevertheless, many sequence reads either do not pass the threshold (quality score: >7 and/or e-value = 1 × 10^−100^) or are part of genome region other than the one in the offline data base. This explains the differences in total reads obtain during sequencing (Figure 2) and the number of aligned reads (Table 1 and Table 2).

Compared to PCR or isothermal amplification assays, the sequencing method is not limited to specific species as any desired animal species can be easily included in the Flesh ID database. Amplification dependent assays are designed to specific targets and for each new species of interest, a new assay has to be developed. Moreover, performing several amplification assays for several animal species of interest is time-consuming. The only drawback of sequencing that the quantification is not possible. 

Many factors can influence the molecular assays for the identification of meat source. The method of cooking, especially pan frying may lead to false negative results [28]. In addition, duration of heat treatment can lead to DNA fragmentation, which will not be advantageous for nanopore sequences, which rely on long DNA reads [29]. 

## 5. Conclusions

In this study, rapid alkaline lysis was combined with nanopore sequencing technology and offline BLAST for the identification of species in meat mixtures in around 4 h. The whole procedure was conducted in a mobile suitcase lab, which facilitates the use at point of need. However, a highly trained person must operate the developed assay and the prices is still high (around 160 Euro per sample). Furthermore, the stability of reagents must be improved to allow long storage at room temperature. In the long run, sequencing will be the standard of molecular diagnostics but data analysis and handling still a great obstacle.

## Figures and Tables

**Table 1:** Number of sequencing hits to each chromosome of eight animal species: The sequencing data presented after 0.5, 1, 3, 9, and 18 h of the high-accuracy and the fast basecalling run were analyzed to identify the time after which all species are correctly detected using chromosome one of sheep, goat, horse, and duck, and chromosome 2 of chicken and cattle, chromosome 3 of turkey, and chromosome 7 of rabbit. The number of hits for each species and the pairwise identity to the reference sequences are shown.

Chromo Some	Species															
	Chicken		Turkey		Goat		Duck		Rabbit		Horse		Cattle		Sheep	
	AC	Hits	AC	Hits	AC	Hits	AC	Hits	AC	Hits	AC	Hits	AC	Hits	AC	Hits
**1**	NC_006088.5	11	NC_015011.2	12	NC_030808.1	174	NC_040046.1	24	NC_013669.1	47	NC_009144.3	64	NC_037328.1	172	NC_040252.1	219
**2**	NC_006089.5	15	NC_015012.2	10	NC_030809.1	170	NC_040047.1	15	NC_013670.1	48	NC_009145.3	26	NC_037329.1	175	NC_040253.1	216
**3**	NC_006090.5	10	NC_015013.2	16	NC_030810.1	160	NC_040048.1	17	NC_013671.1	44	NC_009146.3	34	NC_037330.1	147	NC_040254.1	195
**4**	NC_006091.5	3	NC_015014.2	4	NC_030811.1	153	NC_040049.1	11	NC_013672.1	28	NC_009147.3	26	NC_037331.1	148	NC_040255.1	151
**5**	NC_006092.5	7	NC_015015.2	6	NC_030812.1	158	NC_040050.1	11	NC_013673.1	25	NC_009148.3	24	NC_037332.1	152	NC_040256.1	154
**6**	NC_006093.5	4	NC_015016.2	6	NC_030813.1	141	NC_040051.1	4	NC_013674.1	23	NC_009149.3	25	NC_037333.1	143	NC_040257.1	149
**7**	NC_006094.5	4	NC_015017.2	5	NC_030814.1	164	NC_040052.1	6	NC_013675.1	51	NC_009150.3	25	NC_037334.1	153	NC_040258.1	144
**8**	NC_006095.5	6	NC_015018.2	4	NC_030815.1	154	NC_040053.1	11	NC_013676.1	30	NC_009151.3	29	NC_037335.1	166	NC_040259.1	153
**9**	NC_006096.5	2	NC_015019.2	1	NC_030816.1	145	NC_040054.1	7	NC_013677.1	36	NC_009152.3	28	NC_037336.1	153	NC_040260.1	152
**10**	NC_006097.5	3	NC_015020.2	5	NC_030817.1	148	NC_040055.1	7	NC_013678.1	24	NC_009153.3	27	NC_037337.1	157	NC_040261.1	137
**11**	NC_006098.5	3	NC_015021.2	3	NC_030818.1	145	NC_040056.1	6	NC_013679.1	35	NC_009154.3	14	NC_037338.1	158	NC_040262.1	121
**12**	NC_006099.5	3	NC_015022.2	3	NC_030819.1	138	NC_040057.1	6	NC_013680.1	30	NC_009155.3	13	NC_037339.1	135	NC_040263.1	131
**13**	NC_006100.5	1	NC_015023.2	3	NC_030820.1	134	NC_040058.1	5	NC_013681.1	34	NC_009156.3	12	NC_037340.1	145	NC_040264.1	135
**14**	NC_006101.5	1	NC_015024.2	2	NC_030821.1	149	NC_040059.1	8	NC_013682.1	43	NC_009157.3	40	NC_037341.1	140	NC_040265.1	132
**15**	NC_006102.5	0	NC_015025.2	1	NC_030822.1	133	NC_040060.1	9	NC_013683.1	29	NC_009158.3	30	NC_037342.1	145	NC_040266.1	146
**16**	NC_006103.5	2	NC_015026.2	2	NC_030822.1	134	NC_040061.1	5	NC_013684.1	32	NC_009159.3	24	NC_037343.1	148	NC_040267.1	140
**17**	NC_006104.5	3	NC_015027.2	0	NC_030824.1	124	NC_040062.1	4	NC_013685.1	41	NC_009160.3	27	NC_037344.1	134	NC_040268.1	135
**18**	NC_006105.5	2	NC_015028.2	0	NC_030825.1	130	NC_040063.1	4	NC_013686.1	29	NC_009161.3	42	NC_037345.1	128	NC_040269.1	142
**19**	NC_006106.5	2	NC_015029.2	5	NC_030826.1	123	NC_040064.1	3	NC_013687.1	26	NC_009162.3	20	NC_037346.1	129	NC_040270.1	136
**20**	NC_006107.5	1	NC_015030.2	1	NC_030827.1	136	NC_040065.1	3	NC_013688.1	21	NC_009163.3	16	NC_037347.1	140	NC_040271.1	122
**21**	NC_006108.5	0	NC_015031.2	2	NC_030828.1	142	NC_040066.1	4	NC_013689.1	18	NC_009164.3	21	NC_037348.1	138	NC_040272.1	122
**22**	NC_006109.5	0	NC_015032.2	0	NC_030829.1	130	NC_040067.1	1	Not available	-	NC_009165.3	14	NC_037349.1	129	NC_040273.1	132
**23**	NC_006110.5	1	NC_015033.2	0	NC_030830.1	121	NC_040068.1	2	Not available	-	NC_009166.3	27	NC_037350.1	120	NC_040274.1	142
**24**	NC_006111.5	0	NC_015034.2	2	NC_030831.1	132	NC_040069.1	1	Not available	-	NC_009167.3	16	NC_037351.1	142	NC_040275.1	107
**25**	NC_006112.4	0	NC_015035.2	0	NC_030832.1	109	NC_040070.1	3	Not available	-	NC_009168.3	11	NC_037352.1	110	NC_040276.1	124
**26**	NC_006113.5	1	NC_015036.2	2	NC_030833.1	129	NC_040071.1	3	Not available	-	NC_009169.3	15	NC_037353.1	132	NC_040277.1	114
**27**	NC_006114.5	1	NC_015037.2	0	NC_030834.1	115	NC_040072.1	0	Not available	-	NC_009170.3	18	NC_037354.1	117	Not available	-
**28**	NC_006115.5	0	NC_015038.2	1	NC_030835.1	124	NC_040073.1	0	Not available	-	NC_009171.3	15	NC_037355.1	125	Not available	-
**29**	Not available	-	NC_015039.2	1	NC_030836.1	124	NC_040074.1	0	Not available	-	NC_009172.3	12	NC_037356.1	121	Not available	-
**30**	NC_028739.2	0	NC_015040.2	1	Not available	-	Not available	-	Not available	-	NC_009173.3	9	Not available	-	Not available	-
**31**	NC_028740.2	1	Not available	-	Not available	-	Not available	-	Not available	-	NC_009174.3	13	Not available	-	Not available	-
**32**	NC_006119.4	0	Not available	-	Not available	-	Not available	-	Not available	-	Not available	-	Not available	-	Not available	-
**33**	NC_008465.4	1	Not available	-	Not available	-	Not available	-	Not available	-	Not available	-	Not available	-	Not available	-
**X**	Not available	-	Not available	-	Not available	-	Not available	-	NC_013690.1	33	NC_009175.3	39	NC_037357.1	169	NC_040278.1	170
**W**	NC_006126.5	3	NC_015042.2	1	Not available	-	Not available	-	Not available	-	Not available	-	Not available	-	Not available	-
**Z**	NC_006127.5	10	NC_015041.2	9	Not available	-	NC_040075.1	11	Not available	-	Not available	-	Not available	-	Not available	-
**mt**		0		0		0		0		0		0		0		0

**Table 2:** Results of the identification of the sequencing runs speed. The sequencing data presented after 0.5, 1, 3, 9, and 18 h of the high-accuracy and the fast basecalling run were analyzed to identify the time after which all species are correctly detected. The number of hits for each species and the pairwise identity to the reference sequences are shown.

	High Accuracy Basecalling										Fast Basecalling									
	0.5 h		1 h		3 h		9 h		18 h		0.5 h		1 h		3 h		9 h		18 h	
	Hits	Pairwise Identity (%)	Hits	Pairwise Identity (%)	Hits	Pairwise Identity (%)	Hits	Pairwise Identity (%)	Hits	Pairwise Identity (%)	Hits	Pairwise Identity (%)	Hits	Pairwise Identity (%)	Hits	Pairwise Identity (%)	Hits	Pairwise Identity (%)	Hits	Pairwise Identity (%)
Chicken	0	-	1	84	1	84	3	81.37	4	80.9	0	-	0	-	0	-	2	81.25	2	81.25
Turkey	2	87.5	2	87.5	4	88.28	11	85.96	11	85.96	0	-	1	88.6	2	84.65	10	84	10	84
Duck	2	83.65	2	83.65	2	83.65	17	83.05	22	83.44	1	80.8	3	82.87	5	83.68	16	84.31	19	83.96
Horse	1	79.9	2	79.4	2	79.4	26	80.97	33	80.82	3	83.87	7	82.23	16	82.9	37	82.5	43	82.26
Rabbit	2	87.45	3	88.3	5	88.06	31	84.82	38	85.14	2	85.75	5	86.2	19	85.08	43	84.91	47	84.7
Goat	1	78.4	2	81.4	4	84.46	32	82.26	38	82.3	3	84.33	7	83.7	19	82.95	47	82.93	48	82.94
Cattle	2	87.8	2	87.8	10	82.12	48	82.88	57	83.2	4	80.85	12	81.95	26	82.21	74	81.76	79	81.65
Donkey	3	82.73	5	81.76	13	81.42	62	79.95	80	80.01	1	77.8	16	80.35	54	79.63	110	80.3	114	80.33
Sheep	4	81.05	7	81.96	23	82.94	100	82.94	118	82.93	10	82.96	27	84.35	69	84.1	185	82.95	197	82.93
Pig	124	84.93	189	84.94	405	85.28	2190	84.69	2691	84.8	151	82.28	485	85.35	1337	85.19	3651	84.59	4067	84.47
